# Preparation and Quality Evaluation of Steamed Buns with Spirulina Powder

**DOI:** 10.3390/foods14234136

**Published:** 2025-12-02

**Authors:** Yu Wang, Yingguo Lyu, Longxue Xu, Kunlun Liu, Jie Chen

**Affiliations:** 1 College of Food Science and Engineering, Henan University of Technology, Zhengzhou 450001, China; yuwang373425210@163.com (Y.W.); longxuexu0202@126.com (L.X.); knlnliu@126.com (K.L.); cjie06@163.com (J.C.); 2 Henan Province Wheat-Flour Staple Food Engineering Technology Research Centre, Zhengzhou 450001, China

**Keywords:** spirulina powder, dough microstructure, steamed buns, sensory quality, nutritional value

## Abstract

This study aimed to develop high-quality steamed buns enriched with spirulina powder (SP) to enhance their nutritional value. We investigated the effects of SP addition on the microscopic properties of the dough, as well as the texture, quality, and nutritional profile of the steamed buns. The results indicated that adding more than 5% SP disrupts the gluten network structure, resulting in a reduction in the specific volume of the steamed buns. However, the height-to-diameter ratio of the steamed buns increases slightly with the addition of SP in certain amounts. As the amount of SP added increases, the color of the steamed buns gradually turns green and darkens, while the texture becomes harder. A small amount of SP improves the elasticity and overall sensory score of the steamed buns. With a 2% SP addition, the sensory score of the steamed bun is relatively high; however, overall consumer preference declines when the addition exceeds 5%. SP steamed buns exhibit superior antioxidant properties. The steamed bun containing 5% SP exhibits a total phenolic content (TPC) of 1120.4 mg GAE per 100 g of dry weight, alongside a DPPH radical inhibition rate of 54.78%. These values were 2.21 and 3.51 higher, respectively, compared to buns without SP. Additionally, the protein content of the SP steamed buns increased significantly and the amino acid composition was more comprehensive, with increased levels of lysine and alanine. The results concluded that the steamed buns were of the best quality when 2% SP was added. This study provides a reference for the application of spirulina powder in steamed buns.

## 1. Introduction

Spirulina is a microscopic, filamentous, spiral-shaped cyanobacterium (blue-green alga) that can naturally thrive in alkaline and saline environments, with the ability to double its biomass every 2 to 5 days [1]. The term ‘Spirulina,’ commonly used in trade, marketing, and research alongside its scientific name, refers to a microalga belonging to the genus Limnospira, previously known as Arthrospira [2]. This organism is distinguished by its high economic value, rapid growth rate, substantial organic matter content, and remarkable adaptability to diverse complex environments. Globally, especially in developed countries such as the United States, Germany, and France, spirulina-related products have been extensively integrated into various aspects of daily life, serving consumers in multiple ways, including as sports drinks and beauty cosmetics [3,4].

The World Health Organization (WHO) has approved spirulina as a safe and nutritious high-quality food, with NASA also recognizing its potential use for space missions [5,6]. In particular, spirulina is noteworthy for its rich protein levels (70 g/100 g dw), which are higher than those of meat and legumes [7]. In addition to protein, spirulina is a rich source of vitamins, minerals, and bioactive compounds, including carotenoids, chlorophylls, and phycocyanin, which possess antioxidant properties. This makes spirulina a significant component in combating oxidative stress and supporting overall health. When added to food, it enhances the nutritional value and health benefits of the products [8]. Spirulina has emerged as one of the leading trends in the food industry [9]. Currently, the application of spirulina products is expanding across various categories, including dairy products [10,11,12], biscuits [13], beer [14,15], jelly [16], vegan salad [17], gummy candies [18], green tea [19], etc. Mohammad et al. reported that the addition of 1% and 1.5% SP to halloumi cheese significantly increased the mineral content (P, K, S, Ca, and Mg) [20]. In Ricotta cheese production, the application of SP resulted in increased protein, fat, ash, fibers, carbohydrates, and mineral contents, while imparting a pleasant green color [21].

The application and development of spirulina foods mainly focus on practical snack foods, dairy products, and beverages [10,11,12,13,14,15]. In recent years, the application of flour-based staple food products has gradually garnered attention as a solution to the issues of reliance on single raw materials and unbalanced nutritional components. Rocío Peñalver et al. developed functional gluten-free breads with non-encapsulated spirulina and encapsulated spirulina [22]. The results indicated that free spirulina increased the nutritional properties of the bread, increasing the content of protein, total folate, 9t, 11t-C18:2 (CLA), C22:6n-3 (DHA), and minerals and decreasing the carbohydrate content. The incorporation of SP into pasta also increased the content of unsaturated fatty acids and essential amino acids [23]. Brownies enriched with spirulina showed higher antioxidant capacity and higher phenolic compounds than the control [24].

Steamed buns (Mantou), a significant staple in modern Chinese dietary culture, have undergone thousands of years of evolution and development, becoming a major food source for the Chinese population, particularly in northern regions. Traditionally, the main ingredient of white steamed buns is wheat flour, which primarily provides carbohydrates (approximately 50–60% of dry weight) and protein (about 8–10% of dry weight), though it lacks the first limiting amino acid, lysine, and contains only a minimal amount of other nutrients. Furthermore, advancements in the refining process of wheat flour have led to a significant reduction in vitamins, dietary fiber, and minerals. Consequently, the nutrient deficiencies in wheat flour can lead to long-term imbalances in human nutrition [25]. As a staple food, steamed buns typically offer a limited nutritional profile that fails to meet consumers’ demands for diverse nutrition.

Therefore, numerous studies have focused on increasing the steamed bun diversity and improving the nutritional value by diversifying their raw materials. Examples include Seeds of Coix lacryma-jobi (SCL) steamed buns [26], Kappachychus alvarezii steamed buns [27], tea polyphenols steamed buns [28], potato steamed buns [29], and Siraitia grosvenorii seed flour steamed buns [30]. These studies primarily concentrate on improving the nutritional content of steamed buns. As a nutrient-rich food ingredient, spirulina has the potential for application in steamed buns.

Studies have proven that spirulina has great potential in the application of staple food [22,23,31,32], presenting a viable strategy to address the escalating demand for nutrient diets—innovation that aligns with global trends in functional food development. However, there is currently a lack of systematic investigations on the application of spirulina in steamed bun production and its effect on the quality, organoleptic properties, and nutritional value of the finished products.

The current study aims to explore the effects of varying amounts of spirulina powder (SP) on the microstructure of dough, as well as on the physicochemical indicators, sensory properties, and functional and nutritional properties of steamed buns.

## 2. Materials and Methods

### 2.1. Materials

Xiangmanyuan Special Grade One Wheat Flour (11.2% protein, 38.4% wet gluten, 0.56% ash) was procured from Yihai Kerry Food Industry Co., Ltd. (Shanghai, China). The spirulina (spirulina platensis, 177 μm, corrected by the selected 80-mesh size) was obtained from Etuoke Banner Derong Algae Industry Co., Ltd. (Etuoke, China). High-activity dry yeast was purchased from Angel Yeast Co., Ltd. (Yichang, China). Brilliant green was purchased from the Shanghai Macklin Biochemical Co., Ltd. (Shanghai, China). Iodine, methanol, and anhydrous ethanol were purchased from the Shandong Baiqian Chemical Co., Ltd. (Weifang, China). α-amylase, glucoamylase, Folin phenol reagent, and gallic acid were purchased from the Beijing Solarbio Technology Co., Ltd. (Beijing, China). DPPH reagent was purchased from the Shanghai Yuanye Biology Science and Technology Co., Ltd. (Shanghai, China). All procured chemicals were of analytical grade.

### 2.2. Preparation of Dough and Steamed-Bun-Making Procedure

The basic formulation of dough consists of wheat flour, yeast, and water. Wheat flours were replaced with SP at mass fractions of 1.0%, 2.0%, 3.0%, 4.0%, 5.0%, and 6.0% and mixed thoroughly to ensure uniformity in the mixed flour. A total of 100 g of mixed flour and 0.8 g of yeast were weighed, and the amount of water added was calculated based on 98% of the optimal water absorption rate of the mixed flour measured by a mixograph. The AACC 54-10.02 [33] standard was adopted for the mixograph test, and the optimal water absorption rate was identified when the height of the peak centerline of the blending curve was approximately 50%. It was concluded that a water content of 98% of the optimal value measured by the mixograph would yield suitable dough rheological properties for steamed bun processing, as determined through multiple trials and comparisons. The dry yeast was activated using water at 37 °C for 3 min. Subsequently, the mixed flour, yeast slurry, and water were combined in a dough mixer and kneaded (JHMZ200, Beijing Dongfu Jiuheng Instrument Technology Co., Ltd., Beijing, China) at medium speed for 3 min. The dough was then divided into 50 g portions for future use. After rounding and shaping, the dough was fermented in a constant-temperature humidity incubator (LHS-100CL, Yiheng Scientific Instruments Co., Ltd., Shanghai, China) at 37 °C under 75% relative humidity for 40 min. Finally, the dough was steamed on a tray over boiling water for 20 min and allowed to cool to room temperature, resulting in the final product—spirulina steamed buns. Six samples were prepared for each formulation.

### 2.3. Observation on Microstructure of Dough

The microstructure of the dough was prepared following the method described by Li et al. [34] with modifications. The dough, as detailed in Section 2.2, was frozen at −40 °C for 2 h. Subsequently, it was sliced into thin transparent sheets (approximately 15 μm thick and 5 μm in both length and width) using a knife and spread onto a glass slide. The dough pieces were stained with 0.1% Brilliant Green for 1 min, followed by an additional staining with Lugol’s iodine solution for 1 min. Finally, a coverslip was placed over the specimen. The sample was subsequently observed and photographed using an optical microscope (E5, Ningbo Shunyu Instrument Co., Ltd., Ningbo, China) at a magnification of 400x.

### 2.4. Basic Quality Characteristics of SP Steamed Buns

#### 2.4.1. Specific Volume

The specific volume of the steamed buns was evaluated using the millet replacement method, in accordance with the Chinese standard GB/T 21118-2007 [35] for Wheat Flour Steamed Buns.

#### 2.4.2. Height to Diameter Ratio

The height-to-diameter ratio of steamed buns was determined following the Chinese standard method GB/T 21118-2007 [35] for Wheat Flour Steamed Buns. This was achieved by measuring the diameter and height using a vernier caliper.

#### 2.4.3. Color Analysis

A 10 mm × 10 mm × 5 mm slice was excised from the central crumb of the steamed bun. After calibrating the instrument, the sample was positioned on the measurement platform. Color was analyzed using a colorimeter (CR-400, Konica Minolta, Inc., Tokyo, Japan), with measurements taken at three different positions. The results were expressed in the Hunter Lab color space values: L* (where 0 represents black and 100 represents white), a* (where −60 represents green and +60 represents red), and b* (where −60 represents blue and +60 represents yellow. Here, L*, a*, and b* represented the sample values, while *L*0, *a*0, and *b*0 represented the values of blank samples. The total color change (ΔE) induced by the addition of the SP components was calculated according to the CIE equation:

(1) $$\Delta E\;=\;\sqrt{{\left(L^{*}-L0\right)}^{2}+{\left(a^{*}-a0\right)}^{2}+{\left(b^{*}-b0\right)}^{2}}$$

#### 2.4.4. Textural Properties

After the steamed buns were cooled for 1 h, the textural properties were measured using a TA-XT Plus texture analyzer (PHS-25, Stable Micro Systems, Ltd., Surrey, UK). The testing conditions were as follows: sample slice thickness was 20 mm; probe type was P/35; pre-test speed was 1.0 mm/s; test speed was 1.0 mm/s; post-test speed was 1.0 mm/s; compression strain was 50%; time interval between two compressions was 2 s; and trigger force was 10 g. Each sample was tested in triplicate, and the average value was calculated.

### 2.5. Sensory Analysis

The sensory evaluation of the steamed buns was carried out within 5 h after steaming. Samples were given to ten trained panelists (5 females and 5 males, age range of 20–30) who were asked to evaluate the exterior, structure, taste, and flavor. The scoring method for the sensory evaluation was based on the Chinese standard method GB/T 17320-2013 [36] and Li et al. [34], with certain modifications, as outlined in. The panelists evaluated the samples based on the following characteristics: Specific Volume (20 points), Exterior (20 points), Structure (20 points), Taste (20 points), and Flavor (20 points), culminating in a total score of 100 points, as illustrated in Table 1.

### 2.6. Antioxidant Activity of SP Steamed Buns

#### 2.6.1. Preparation of Extract

A precisely weighed sample of 2 g of freeze-dried steamed buns (precision: ±0.0001 g) was placed into a centrifuge tube. Subsequently, 20 mL of 75% (*v*/*v*) anhydrous ethanol was added to the tube. The mixture was vortex-mixed thoroughly and incubated at room temperature for 30 min to ensure complete extraction. The mixture was then centrifuged at 5000 rpm for 20 min, and the supernatant was collected as the test solution for subsequent analysis [37].

#### 2.6.2. DPPH Free Radical Scavenging Ability

A 2 mL aliquot of the sample extract was added to 2 mL of a DPPH standard solution (0.1 mM in ethanol) and 2 mL of 75% anhydrous ethanol. The resulting mixture was vortexed thoroughly and incubated in the dark at room temperature for 30 min. The absorbance of the mixed solution was measured at 517 nm using a spectrophotometer (7230G, Shanghai Shun Yu Heng Ping Scientific Instrument Co., Ltd., Shanghai, China), with anhydrous ethanol serving as the blank reference [37].

In this study, the absorbance of three different mixtures was measured to evaluate the antioxidant activity of the sample extracts. The first mixture, denoted as *A*1, consisted of 2 mL of the sample extract combined with 2 mL of DPPH solution. The second mixture, referred to as *A*2, included 2 mL of the sample extract and 2 mL of ethanol, which served as a background correction to account for any color contributed by the sample itself. Finally, *A*0 represented the absorbance of a control mixture composed of 2 mL of ethanol and 2 mL of DPPH solution. The DPPH free radical scavenging activity of the sample was calculated using Equation (1). This methodology allowed for a clear assessment of the antioxidant capacity by isolating the contributions from the sample extract and ensuring accurate readings.

(2) $$\mathrm{Scavenging}\;\mathrm{activity}\;(\%)=[1-\frac{\left(A2-A1\right)}{A0}]\;\times \;100$$

### 2.7. Total Phenolic Content of SP Steamed Buns

The total phenolic content (TPC) in the phenolic extracts was determined using the Folin–Ciocalteu method [38]. Freeze-dried steamed bun powder (0.5 g) was mixed with an 80% methanol solution (8 mL), and the mixture was subjected to ultrasonication at 40 kHz and 60 °C for 20 min to facilitate the reaction. Following ultrasonication, the mixture was centrifuged at 5000 rpm for 20 min, and the supernatant was collected as the test solution. A standard curve was constructed, with the gallic acid concentration (x, mg/mL) plotted on the *x*-axis and absorbance (y) on the *y*-axis. The regression equation is Y = 13.7X + 0.2608, R^2^ = 0.99654. An aliquot of 0.2 mL of the extract, which was replaced by anhydrous ethanol as the blank reference sample, was accurately measured into a test tube. Subsequently, 3 mL of 7.5% (g/mL) Na_2_CO_3_ and 1 mL of the Folin–Ciocalteu reagent were added. The mixture was then reacted for 50 min at 50 °C in a constant-temperature shaker, shielded from light. The absorbance of the samples was read at 760 nm in a spectrophotometer (7230G, Shanghai Shun Yu Heng Ping Scientific Instrument Co., Ltd., Shanghai, China), and the TPC was calculated as milligrams of gallic acid equivalents (GAE) per gram of sample (mg GAE/g). All measurements were performed in triplicate.

### 2.8. Determination of Protein Content

The total nitrogen content in the steamed buns was determined using the Kjeldahl method. The nitrogen content of steamed buns was determined using an automatic Kjeldahl analyzer (KieltecTM8400, Foss, Hillerød, Denmark).

### 2.9. Total Free Amino Acids (TFAAs) Content of SP Steamed Buns

The TFAA content was analyzed using a Fully Automated Amino Acid Analyzer (Sykam S433D, Sykam GmbH, Gräfelfing, Germany) with an ion exchange chromatographic column [39]. We accurately weighed 90–100 mg of freeze-dried steamed buns powder (precision: ±0.0001 g), ensuring that the total protein content did not exceed 10 mg, into a hydrolysis tube. Then, 10 mL of 6 M hydrochloric acid (HCl) and 3–4 drops of phenol were added to the tube. The tube was flushed with nitrogen gas for 2 min to establish an anaerobic environment. The sample was hydrolyzed in a drying oven at 110 ± 1 °C for 22 h. After cooling to room temperature, the hydrolysate was filtered through qualitative filter paper, and the filtrate was diluted to 50 mL with deionized water. Then, 1 mL of the diluted solution was transferred to a rotary evaporator and dried at 50 °C. The residue was redissolved in 1 mL of deionized water, and the drying process was repeated until complete desiccation was achieved. The final residue was reconstituted in 1 mL of pH 2.2 sample dilution buffer. The solution was sonicated for 2 min and filtered through a 0.22 μm aqueous phase membrane into a liquid vial for analysis. The chromatography conditions were established as follows: injected sample volume of 50 μL; mobile phase consisting of Buffer A, which is a mixture of Trisodium citrate, citric acid monohydrate, and muriatic acid in a ratio of 2:1:1; mobile phase flow rate set at 0.5 mL/min; and detection wavelengths of 570 nm and 440 nm.

### 2.10. Statistics Analysis

All data were expressed as mean ± standard deviation (SD) from three replicates, and Origin 2024 and Image J window64 software were utilized for mapping. One-way analysis of variance (ANOVA) was employed to analyze the data, while Duncan’s multiple range test in SPSS 22.0 was used to assess significance. A probability value of *p* < 0.05 was considered statistically significant.

## 3. Results

### 3.1. The Influence of SP Addition on the Microstructure of Dough

**Figure 1:**
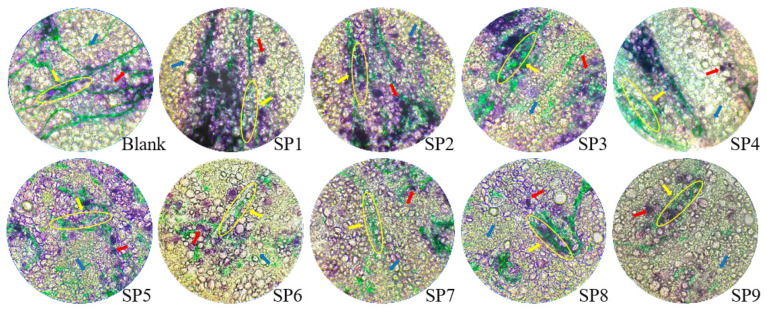
Effect of SP addition on dough microstructure after staining of starch and protein. The amount of SP added in the dough is represented as Blank, SP1, SP2, SP3, SP4, SP5, SP6, SP7, SP8, and SP9, corresponding to blank, 1%, 2%, 3%, 4%, 5%, 6%, 7%, 8%, and 9%, respectively. Yellow refers to gluten network structure; red refers to starch granules; blue refers to spirulina powder.

The gluten network structure of dough is a critical factor that determines its physical properties and the quality of the final product. This network structure notably impacts the springiness, extensibility, and gas-holding capacity of the dough. To visually assess the effect of SP addition on the gluten structure in steamed bun dough, both starch and protein within the dough were stained for observation. The stained starch granules appear purple, the gluten protein is dark green, and SP exhibits a chartreuse color. In the absence of SP, the gluten structure forms a complex and continuous network, with starch granules either wrapped or closely attached to this gluten matrix. With SP addition ranging from 1% to 4%, the dark green gluten network remains continuous and compact, enveloping some starch granules. At SP levels of 5% to 6%, images revealed that some continuous gluten protein network structures persist in the dough (see Figure 1). However, according to Li’s research [34], when the addition of SP exceeds 6%, the gluten network is significantly compromised, resulting in the appearance of ‘holes’. This discrepancy may be attributed to variations in water addition; Li’s method incorporated approximately 30% water during dough preparation [34], whereas this experiment utilized around 55%. Increased water addition facilitates gluten network formation. Therefore, although dietary fiber competes with gluten protein for water, sufficient water remains available for gluten protein hydration, promoting a stable gluten network structure. In scenarios with low water addition, the competition for water by spirulina is more pronounced, hindering gluten network formation. Conversely, in high water addition scenarios, this competition is less significant, and the damage to the gluten network primarily results from the dilution effect of SP on gluten protein.

When the addition of SP increases to 7% to 9%, the gluten protein network structure becomes loose and disordered, resulting in the formation of ‘nodular’ shapes. The concentration of starch granules diminishes, with some granules no longer enveloped by the gluten network. This phenomenon may arise because the increased spirulina hinders the formation of original disulfide bonds within the dough, adversely affecting the gluten network structure and promoting the depolymerization of larger protein molecules into smaller ones. Consequently, damage to the gluten network can lead to the unwrapping and destabilization of starch granules, which damages the dough and gluten structure, significantly reducing the structural stability of the dough. Fan et al. also pointed out that exceeding a certain threshold causes competition between SP-MICs and gluten proteins for water binding or for occupying cross-linking sites between gluten proteins, thereby disrupting the original protein network [32].

### 3.2. The Effects of SP Addition on Basic Quality Characters of Steamed Buns

#### 3.2.1. Specific Volume and Height-to-Diameter Ratio of SP Steamed Buns

**Figure 2:**
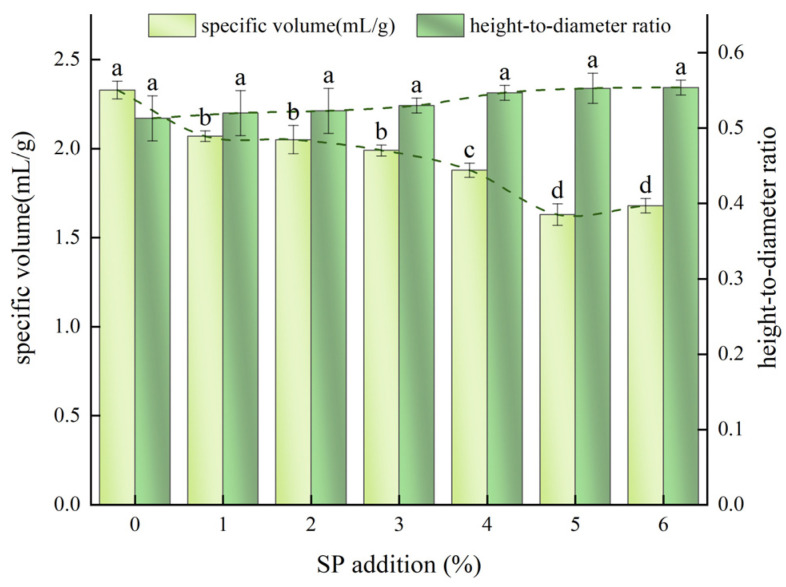
Effect of SP addition on specific volume and height–diameter ratio of steamed buns. Data with different lowercase letters indicate significant differences among the steamed buns with different SP addition, respectively (*p* < 0.05).

The specific volume and height-to-diameter ratio of steamed buns are critical indicators for assessing their quality. An increase in the addition of SP generally correlates with a decline in the specific volume of steamed buns. Specifically, when the SP addition ranges from 1% to 3%, the specific volume experiences a slight decrease, indicating that a modest amount of SP has a relatively minor effect on the gluten protein structure. However, when the SP addition exceeds 3%, a significant reduction in specific volume occurs (as illustrated in Figure 2). This phenomenon may be attributed to the dilution of gluten protein, which weakens the gluten network structure and diminishes the gas-holding capacity of the dough. Consequently, during the fermentation process, gases are less effectively retained, leading to a reduction in internal gas spaces within the steamed buns post-steaming, resulting in a denser dough structure and a notable decrease in both volume and specific volume.

Conversely, as the amount of SP added increases, Figure 2 demonstrates a general upward trend in the height-to-diameter ratio of the steamed buns. The incorporation of SP positively influences the height-to-diameter ratio of the steamed buns. This effect may be attributed to the compromised gluten structure resulting from the addition of spirulina, which is unable to fully retain gas [40]. Consequently, bubbles tend to accumulate at the top, promoting vertical growth of the steamed bread rather than lateral expansion.

#### 3.2.2. Color Change of SP Steamed Buns 

The effect of SP on the color properties of steamed buns is illustrated in Table 2. Color is a crucial characteristic of steamed buns, significantly influencing consumer preferences for the product. Golden tone (L value 55–60, A value 5–8) is most in line with consumers’ cognitive expectation of traditional steamed bun. The steamed buns prepared with SP exhibited a darker hue, as indicated by the lower L* values, alongside reduced redness (lower a* values) and diminished yellow tones (lower b* values). The L*, a*, and b* values of the steamed buns were significantly decreased with the addition of SP (*p* < 0.05). The color changes (ΔE) were highly correlated with SP content (*p* < 0.05). As the amount of SP increased, the color of the steamed buns transitioned from light green to green and ultimately to dark green. This change is primarily attributed to the presence of chlorophyll and C-phycocyanin pigments in spirulina [31]. Traditional steamed buns are characterized by their milky white color. In recent years, consumer attitudes have shifted, leading to a rise in the popularity of multigrain steamed buns and colorful fruit and vegetable variants. The vibrant colors of SP steamed buns may have a distinct appeal to certain consumers, and the natural pigments of spirulina can replace synthetic pigments to make natural-colored steamed buns, which gratify the requirements of a clean label.

#### 3.2.3. Textural Properties of SP Steamed Buns

Texture is a crucial attribute of steamed buns for consumers. Variations in the texture data of steamed buns may serve as quality indicators for subsequent consumer evaluations. The evaluated textural properties of the steamed buns are presented in Table 3. Desirable characteristics for steamed bun products include optimal values of hardness and springiness. The hardness values of the steamed buns increased significantly from 389 g to 1059 g following the addition of SP, with this increase being statistically significant (*p* < 0.05). This increase can be attributed to the dietary fiber in SP, which absorbs water and expands, thereby enhancing the firmness of the steamed buns. Aydoğdu et al. indicated that cakes became harder with an increase in dietary fiber content [41]. Similarly, Joshi et al. demonstrated a positive correlation between the hardness of biscuits and the dietary fiber content, particularly as the amount of wheat flour is replaced with whole wheat flour and gram flour [42]. Chewiness, defined as the energy required to disintegrate food, is a parameter that depends on hardness. Both hardness and chewiness exhibit a step-like increase; when the addition of SP reaches 5% to 6%, the hardness and chewiness become excessively high, resulting in a rough texture that is difficult to chew, thereby detracting from the overall sensory experience of the steamed buns.

This phenomenon may be attributed to the interaction between the protein in spirulina and the protein in the dough, which involves hydrogen bonding between amide-hydroxyl and hydroxyl-carbonyl groups with polar compound groups. Such bonding can increase the hardness and resistance of the dough [31].

According to Table 3, the springiness of steamed buns increased after the addition of SP compared to the blank group, while cohesiveness exhibited an overall downward trend. The resilience of the steamed buns initially increased before declining. The results suggest that adding a small amount of SP can enhance the resilience and overall quality of the steamed buns. Fan et al. also pointed out that the elasticity and viscosity of SP-MIC noodles increased and then decreased with the addition of spirulina, reaching a maximum value at M3 [32].

### 3.3. Overall Sensory Quality of SP Steamed Buns

**Figure 3:**
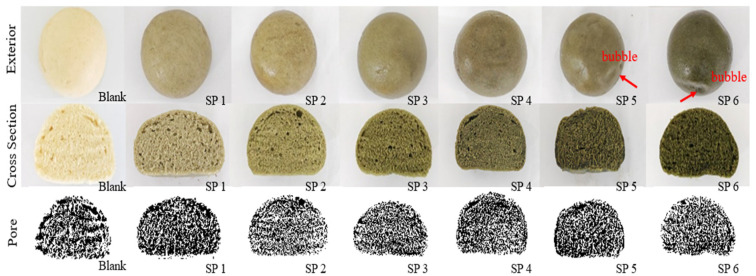
The appearance, cross-section, and pores of SP steamed buns. The amount of SP added to the steamed buns is represented as Blank, SP1, SP2, SP3, SP4, SP5, and SP6, corresponding to 0%, 1%, 2%, 3%, 4%, 5%, and 6%, respectively.

According to the observations presented in Figure 3, an increase in the amount of SP results in a gradual darkening of the color of the steamed buns and a significant reduction in their volume. Furthermore, the surface texture becomes increasingly rough, with the appearance of small spots or bubbles. When the addition of SP reaches between 5% and 6%, the steamed buns exhibit asymmetrical shapes and noticeable depressions. This alteration may be attributed to the effect of spirulina on the dough’s extensibility and surface smoothness, ultimately leading to a rougher surface during the steaming process.

During the preparation of steamed buns, gluten proteins undergo cross-linking to establish a three-dimensional network. The carbon dioxide produced by yeast during fermentation creates fine pores within the dough, imparting a loose and porous structure to the buns [43]. As illustrated in Table 4, an increase in the addition of SP correlates with a rising average area of the pores in the cross-section of the steamed buns, while the stomatal density shows a declining trend. The incorporation of spirulina dilutes the gluten proteins and compromises the formation of the gluten network, leading to smaller pores in the cross-section and a denser structure. The relatively high porosity, coupled with improved gas retention, suggests that the pores are small and uniform, thereby improving the overall tissue structure.

**Figure 4:**
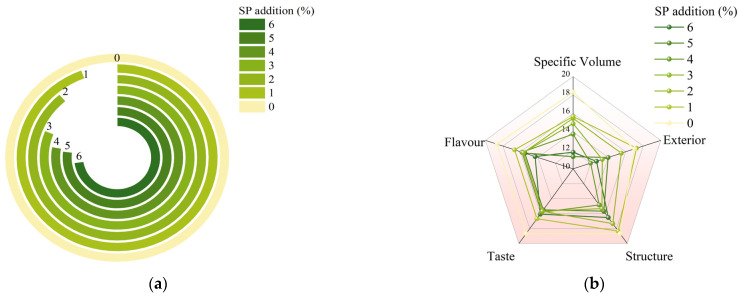
Effect of SP on sensory quality of steamed buns. (**a**) Total score of sensory evaluation of steamed buns; (**b**) Radar chart of sensory evaluation of steamed buns.

The sensory evaluations of the steamed buns are illustrated in Figure 4b. An increase in the proportion of SP resulted in significant differences in specific volume, exterior, structure, taste, flavor, and overall acceptability of the steamed buns (*p* < 0.05). When the SP addition ranged from 1% to 3%, the steamed buns were endowed with the unique color and flavor of spirulina. This is attributed to the proteins and amino acids present in SP, which can undergo a Maillard reaction with the metabolic products of yeast during fermentation. This reaction leads to the formation of volatile flavor compounds such as pyrazines and aldehydes, contributing unique flavors to the steamed buns. However, when the SP content exceeded 3%, the flavor of the steamed buns deteriorated, and the taste was accompanied by a fishy flavor. As the SP content increased, the total score of the steamed buns exhibited a downward trend, with sensory scores decreasing (Figure 4a). These results suggest that a partial replacement of wheat flour with 2% SP in steamed buns yields optimal physicochemical indicators and overall acceptability. The lighter green color enhances consumer acceptance, as it is perceived as a healthy and nutritious characteristic [44]. Mamat et al. [27] observed a similar phenomenon in their investigation of the effects of seaweed composite flour on the quality of steamed buns. The greenness and yellowness values of the steamed buns increased with a higher percentage of seaweed composition. In their sensory analysis, formulation F1 received a higher preference rating from panelists compared to other formulations due to its lower concentration of seaweed powder (1.5%). Conversely, Zhou et al. [30] noted that the sliced area and brightness of steamed bread gradually decreased with the addition of Siraitia grosvenorii seed flour. Additionally, the stomatal contrast, stoma number, porosity, and stomatal density exhibited an initial increase followed by a subsequent decrease.

### 3.4. Correlation Analysis Between the Quality Characteristics of SP Steamed Buns

**Figure 5:**
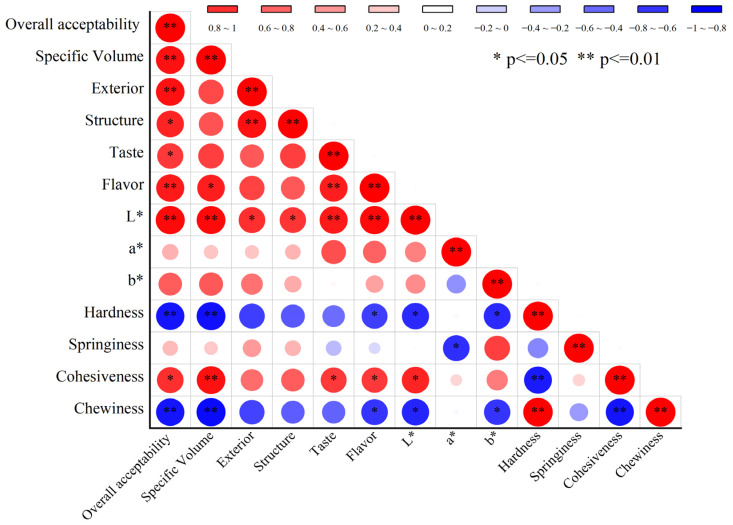
Correlation analysis between the quality characteristics of SP steamed buns * significant at 0.05 level; ** significant at the level of 0.01 (extremely significant).

As illustrated in Figure 5, the overall acceptability of steamed buns is significantly positively correlated (*p* < 0.01) with their specific volume, exterior, flavor, and lightness (L*), while it is also significantly positively correlated (*p* < 0.05) with their structure and cohesiveness. Conversely, a significant negative correlation (*p* < 0.01) exists between acceptability and both hardness and chewiness. Additionally, the specific volume of steamed buns demonstrates a significant negative correlation (*p* < 0.01) with hardness and chewiness. Furthermore, the exterior of the steamed buns is significantly positively correlated (*p* < 0.01) with their structure and positively correlated (*p* < 0.05) with lightness (L*). The taste of steamed buns is significantly positively correlated (*p* < 0.01) with both flavor and lightness (L*) and positively correlated (*p* < 0.05) with cohesiveness. Moreover, the lightness (L*) of steamed buns is significantly negatively correlated (*p* < 0.05) with hardness and chewiness. Hardness shows a significant positive correlation (*p* < 0.01) with chewiness, while it exhibits a significant negative correlation (*p* < 0.01) with cohesiveness. Lastly, cohesiveness is significantly negatively correlated (*p* < 0.01) with chewiness. The acceptability of steamed buns to consumers largely depends on their specific volume, exterior, and taste. Steamed buns with a larger specific volume tend to be softer and more flavorful. The condition of the epidermis, color, and aroma of steamed buns directly influence consumers’ first impressions. Steamed buns characterized by smooth, even skin, bright color, and a fermented fragrance are considered more acceptable.

### 3.5. The Antioxidant Capacity of SP Steamed Buns

**Figure 6:**
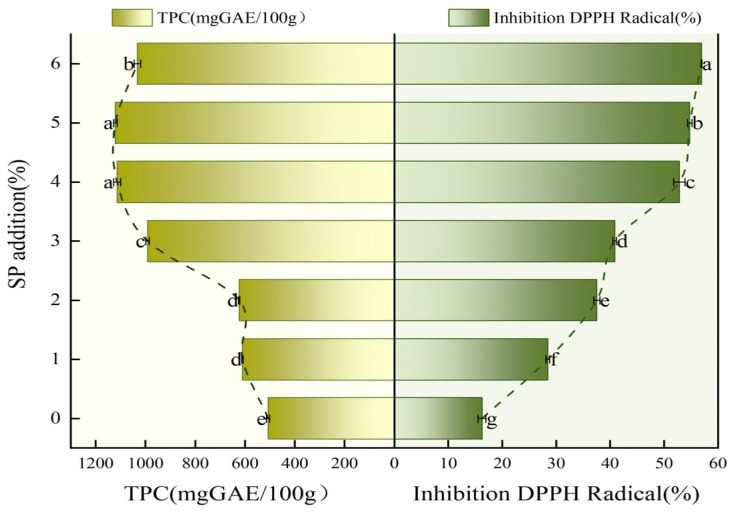
Effect of SP on TPC and antioxidant capacity of the steamed buns. Data with different lowercase letters indicate significant differences among the steamed buns with different SP additions, respectively (*p* < 0.05).

The total phenolic content (TPC) and antioxidant capacity of the steamed bun samples were significantly enhanced by substituting the flour with SP, with these increases being statistically significant (*p* < 0.05), as illustrated in Figure 6. The steamed buns prepared with 5% SP exhibited the highest TPC at 1120.4 mg GAE/100 g dry weight, while those with 6% SP demonstrated the maximum antioxidant activity, achieving a 56.96% inhibition rate. The TPC values and the percentage inhibition of DPPH radicals in the steamed buns containing 5% SP were 2.21 and 3.51 times greater, respectively, compared to those without SP. Ismail et al. also stated that Ricotta cheese fortified with 1% spirulina powder had significantly higher total phenolic content (7.03 ± 0.08 mg GAE/g) and antioxidant capacity (20.27 ± 0.80%) compared to that in control Ricotta cheese (5.11 ± 0.07 mg GAE/g and 15.25 ± 0.41%), respectively [21]. The enhanced antioxidant properties of the steamed buns can be attributed to the antioxidant and carotenoid compounds present in SP, particularly notable for β-carotene, phycocyanin, zeaxanthin, cryptoxanthin, lutein, and superoxide dismutase (SOD) [45]. Furthermore, spirulina is abundant in various phenolic compounds, including phenolic acids, flavonoids, and tannins, all of which exhibit significant antioxidant activities that effectively scavenge free radicals and inhibit lipid oxidation. Previous studies have indicated a positive correlation between phenolic content and antioxidant activity [46].

### 3.6. The Protein Content and Composition of Free Amino Acids in SP Steamed Buns

The protein concentrations of steamed bun from groups blank to SP6 are illustrated in Table 5. The blank group exhibited a protein content of 9.61%, while the protein levels in SP1 to SP6 increased progressively to 10.23%, 10.69%, 11.23%, 11.63%, 12.09%, and 12.82%, respectively. This effect can be attributed to the spirulina, which is highly protein-rich, with protein content accounting for up to 70% of its dry weight [47]. Protein content analysis revealed that the addition of 1%to 6% SP significantly enhanced the protein content in steamed buns. However, despite the contribution from spirulina protein, wheat proteins remained the predominant component of the total protein content in SP steamed buns. However, the protein in wheat flour is relatively low in essential amino acids, particularly lysine and methionine, resulting in a lower biological value compared to animal protein. Spirulina, recognized as a nutrient-rich superfood, possesses an amino acid profile that closely aligns with human nutritional requirements, particularly due to its high concentration of essential amino acids. Furthermore, the amino acids in spirulina are available in both free form and as small peptides, which facilitates their digestion and absorption by the human body.

As demonstrated in Table 5, an increase in the addition of SP significantly enhances the total amino acid and essential amino acid content in steamed buns. Specifically, when the SP addition reached 6%, the levels of lysine, alanine, and total essential amino acids in the steamed buns increased by 79.72%, 118.29%, and 57.53%, respectively, compared to the control group. According to Montevecchi et al. [48], in baked Spirulina-enriched products, the amino acid concentrations of histidine, threonine, tyrosine, isoleucine, leucine, phenylalanine, and lysine were significantly higher than the control.

## 4. Conclusions

This study systematically investigates the effects of incorporating spirulina powder (SP) on the quality and nutritional properties of steamed buns. The results demonstrated that in scenarios involving elevated water addition, the impairment to the gluten network is predominantly attributable to the dilution effect of SP on gluten protein, thereby exerting a consequential influence on the characteristics of steamed buns. The incorporation of SP results in an augmentation of the height–diameter ratio of steamed buns, conferring upon them a natural green and distinctive flavor profile. Health-promoting compounds, such as protein content, free amino acids, and antioxidant activity, in steamed buns formulated with partial substitution of SP are found to be significantly enhanced at higher levels of SP. The total phenolic content (TPC) value and DPPH radical inhibition rate of steamed buns with 5% SP addition are 2.21 times and 3.51 times higher, respectively, in comparison to buns without SP. Furthermore, when the SP addition reached 6%, the levels of protein content, lysine, alanine, and total essential amino acids in the steamed buns increased by 3.21%, 79.72%, 118.29%, and 57.53%, respectively, in comparison to the control group. It is evident that superior specific volume, springiness, and color, in addition to the optimal organoleptic properties, are achieved with a 2% addition of SP. The light green color of SP-enriched steamed buns is likely to enhance consumer acceptability due to the perceived health and nutritional benefits. Consequently, in order to produce steamed buns with the desired quality and sensory properties, as well as increased functional components and antioxidant activity, SP can be effectively incorporated into steamed bun formulations. Subsequent research will integrate dietary fiber, minerals, and proximate compositions to further evaluate the application value of SP to steamed buns.

## Figures and Tables

**Table 1:** Scoring method for sensory evaluation of steamed buns.

Parameters		Score	Scoring Standard
Specific Volume (mL/g)		20	Specific volume higher than 2.5 mL/g is defined as the full score; for every 0.1 mL/g decrease, 1 point is deducted.
Exterior	Surface Color	10	The evaluation criteria for the visual quality of the samples are categorized as follows: Light green, uniform, and glossy samples score between 8.1 and 10 points. Samples that are green, moderately uniform, and exhibit noticeable gloss are rated between 6.1 and 8 points. Conversely, samples that are dark green, uneven, dull, and lack gloss receive scores ranging from 1 to 6 points. This classification allows for a clear and systematic assessment of the samples’ visual characteristics.
	Surface Smoothness	10	The evaluation criteria for the surface smoothness are categorized as follows: a score of 8.1 to 10 points indicates a smooth, wrinkle-free, symmetrical, and firm condition. A score ranging from 6.1 to 8 points reflects moderate smoothness. Conversely, a score between 1 and 6 points denotes roughness, the presence of hard spots, or asymmetry.
Structure		20	The evaluation criteria for pore uniformity and resilience are categorized as follows: Fine, uniform pores exhibiting excellent resilience upon finger compression are rated between 16.1 and 20 points. Moderately uniform pores receive a score ranging from 12.1 to 16 points. In contrast, large, uneven pores that demonstrate poor resilience are assigned a score between 1 and 12 points.
Taste		20	The evaluation of taste can be categorized into three distinct ranges: a non-sticky texture with a slight spirulina flavor, which scores between 16.1 and 20 points; a moderate spirulina flavor that falls within the range of 12.1 to 16 points; and a sticky texture accompanied by an excessive spirulina flavor, which is rated from 1 to 12 points. This classification provides a clear framework for assessing the sensory attributes of spirulina in various applications.
Flavor		20	The evaluation criteria for the aroma and bitterness of the product are categorized as follows: A fresh fermented aroma with no bitterness is rated between 16.1 and 20 points. Mild bitterness is assigned a score ranging from 12.1 to 16 points. In contrast, products exhibiting no fermented aroma but pronounced bitterness are rated between 1 and 12 points.
Total points		100	

**Table 2:** Effect of SP addition on the color of steamed buns.

SP Addition (%)	L*	a*	b*	ΔE
0	82.74 ± 0.10 ^a^	−2.31 ± 0.03 ^a^	16.39 ± 0.10 ^c^	0 ^a^
1	57.55 ± 0.30 ^b^	−5.49 ± 0.04 ^e^	18.46 ± 0.34 ^ab^	25.48 ± 0.32 ^b^
2	51.97 ± 0.54 ^c^	−5.22 ± 0.02 ^d^	18.94 ± 0.15 ^a^	31.01 ± 0.54 ^c^
3	45.64 ± 0.26 ^d^	−5.54 ± 0.06 ^e^	17.47 ± 1.22 ^bc^	37.27 ± 0.24 ^d^
4	40.44 ± 0.12 ^e^	−4.53 ± 0.12 ^c^	16.34 ± 0.41 ^c^	42.36 ± 0.12 ^e^
5	35.59 ± 0.30 ^f^	−3.68 ± 0.14 ^b^	14.82 ± 0.58 ^d^	47.20 ± 0.30 ^f^
6	33.01 ± 0.47 ^g^	−4.70 ± 0.20 ^c^	13.53 ± 0.86 ^e^	49.88 ± 0.47 ^g^

Values in the same column with different letters are significantly different (*p* < 0.05).

**Table 3:** Effect of SP addition on texture characteristics of steamed buns.

SP Addition (%)	Hardness (g)	Springiness	Cohesiveness	Chewiness	Resilience
0	389.258 ± 6.61 ^e^	0.885 ± 0.01 ^c^	0.805 ± 0.00 ^a^	283.676 ± 4.09 ^d^	0.414 ± 0.01 ^c^
1	364.300 ± 14.03 ^e^	0.944 ± 0.00 ^a^	0.795 ± 0.02 ^ab^	307.216 ± 6.94 ^d^	0.430 ± 0.00 ^b^
2	536.474 ± 7.70 ^d^	0.938 ± 0.01 ^a^	0.784 ± 0.00 ^abc^	419.002 ± 23.00 ^c^	0.442 ± 0.00 ^a^
3	631.758 ± 37.52 ^c^	0.920 ± 0.01 ^b^	0.784 ± 0.00 ^abc^	456.290 ± 32.00 ^c^	0.415 ± 0.00 ^c^
4	737.711 ± 33.69 ^b^	0.893 ± 0.00 ^c^	0.787 ± 0.00 ^ab^	507.306 ± 23.85 ^b^	0.420 ± 001 ^c^
5	1057.835 ± 57.36 ^a^	0.890 ± 0.01 ^c^	0.759 ± 0.00 ^c^	724.785 ± 47.35 ^a^	0.398 ± 0.00 ^d^
6	1059.251 ± 25.43 ^a^	0.902 ± 0.01 ^c^	0.775 ± 0.03 ^bc^	740.197 ± 36.59 ^a^	0.411 ± 0.00 ^c^

Values in the same column with different letters are significantly different (*p* < 0.05).

**Table 4:** Stomatal characteristics of SP steamed buns.

SP Addition (%)	Average Stomatal Area/mm^2^	Stomatal Density/(Pieces/cm^2^)	Porosity/%
0	0.44 ± 0.12 ^d^	83.00 ± 7.07 ^a^	35.75 ± 6.74 ^b^
1	0.48 ± 0.04 ^cd^	79.00 ± 1.41 ^a^	37.58 ± 2.26 ^b^
2	0.45 ± 0.01 ^d^	79.00 ± 1.41 ^a^	35.22 ± 1.61 ^b^
3	0.76 ± 0.03 ^bc^	62.00 ± 2.83 ^b^	46.95 ± 0.21 ^a^
4	0.93 ± 0.01 ^b^	56.00 ± 0.00 ^b^	52.05 ± 0.50 ^a^
5	1.26 ± 0.28 ^a^	43.00 ± 7.07 ^c^	53.05 ± 3.32 ^a^
6	1.32 ± 0.07 ^a^	40.00 ± 2.83 ^c^	52.66 ± 0.76 ^a^

Values in the same column with different letters are significantly different (*p* < 0.05).

**Table 5:** Protein content and composition of free amino acids in SP steamed buns.

	SP Addition (%)						
	0	1	2	3	4	5	6
Protein	9.61 ± 0.20 ^g^	10.23 ± 0.17 ^f^	10.69 ± 0.17 ^e^	11.23 ± 0.21 ^d^	11.63 ± 0.10 ^c^	12.09 ± 0.31 ^b^	12.82 ± 0.12 ^a^
Asp	0.485 ± 0.01 ^f^	0.535 ± 0.01 ^e^	0.683 ± 0.02 ^d^	0.699 ± 0.01 ^d^	0.720 ± 0.01 ^c^	0.745 ± 0.01 ^b^	0.833 ± 0.00 ^a^
Thr	0.297 ± 0.01 ^e^	0.340 ± 0.01 ^d^	0.366 ± 0.01 ^c^	0.382 ± 0.01 ^b^	0.384 ± 0.01 ^b^	0.374 ± 0.01 ^bc^	0.432 ± 0.01 ^a^
Ser	0.528 ± 0.02 ^c^	0.628 ± 0.01 ^b^	0.629 ± 0.02 ^b^	0.640 ± 0.01 ^b^	0.645 ± 0.0 ^b^	0.628 ± 0.01 ^b^	0.725 ± 0.01 ^a^
Glu	3.659 ± 0.02 ^b^	3.705 ± 0.05 ^b^	4.392 ± 0.22 ^a^	4.400 ± 0.10 ^a^	4.250 ± 0.14 ^a^	3.814 ± 0.02 ^b^	4.430 ± 0.05 ^a^
Gly	0.362 ± 0.00 ^d^	0.376 ± 0.02 ^d^	0.475 ± 0.01 ^b^	0.450 ± 0.02 ^c^	0.499 ± 0.01 ^b^	0.486 ± 0.02 ^b^	0.561 ± 0.00 ^a^
Ala	0.328 ± 0.01 ^f^	0.371 ± 0.01 ^e^	0.548 ± 0.01 ^d^	0.604 ± 0.02 ^c^	0.608 ± 0.01 ^c^	0.651 ± 0.01 ^b^	0.716 ± 0.03 ^a^
Cys	0.969 ± 0.02 ^d^	0.963 ± 0.01 ^d^	1.119 ± 0.05 ^c^	1.231 ± 0.04 ^b^	1.347 ± 0.02 ^a^	1.356 ± 0.03 ^a^	1.372 ± 0.08 ^a^
Val	0.108 ± 0.01 ^c^	0.121 ± 0.03 ^bc^	0.126 ± 0.01 ^abc^	0.138 ± 0.02 ^abc^	0.145 ± 0.02 ^ab^	0.131 ± 0.01 ^abc^	0.155 ± 0.01 ^a^
Met	0.146 ± 0.01 ^c^	0.148 ± 0.02 ^c^	0.191 ± 0.00 ^b^	0.214 ± 0.02 ^a^	0.211 ± 0.01 ^ab^	0.213 ± 0.01 ^a^	0.227 ± 0.02 ^a^
Ile	0.313 ± 0.02 ^e^	0.347 ± 0.01 ^d^	0.434 ± 0.01 ^c^	0.477 ± 0.01 ^b^	0.465 ± 0.01 ^b^	0.474 ± 0.02 ^b^	0.520 ± 0.01 ^a^
Leu	0.682 ± 0.01 ^d^	0.743 ± 0.02 ^d^	0.943 ± 0.02 ^c^	0.998 ± 0.01 ^bc^	1.069 ± 0.09 ^ab^	1.011 ± 0.01 ^bc^	1.118 ± 0.05 ^a^
Tyr	0.209 ± 0.02 ^e^	0.232 ± 0.01 ^d^	0.260 ± 0.01 ^c^	0.280 ± 0.01 ^bc^	0.297 ± 0.01 ^ab^	0.315 ± 0.01 ^a^	0.304 ± 0.01 ^a^
Phe	0.488 ± 0.02 ^e^	0.507 ± 0.01 ^e^	0.565 ± 0.02 ^d^	0.570 ± 0.02 ^cd^	0.599 ± 0.01 ^bc^	0.607 ± 0.02 ^b^	0.704 ± 0.00 ^a^
His	0.277 ± 0.02 ^c^	0.262 ± 0.00 ^c^	0.306 ± 0.01 ^b^	0.307 ± 0.01 ^b^	0.313 ± 0.01 ^b^	0.309 ± 0.01 ^b^	0.361 ± 0.01 ^a^
Lys	0.217 ± 0.01 ^e^	0.236 ± 0.01 ^d^	0.303 ± 0.01 ^c^	0.341 ± 0.02 ^b^	0.342 ± 0.00 ^b^	0.343 ± 0.01 ^b^	0.390 ± 0.01 ^a^
Arg	0.391 ± 0.01 ^d^	0.401 ± 0.01 ^d^	0.498 ± 0.01 ^c^	0.502 ± 0.01 ^c^	0.512 ± 0.01 ^c^	0.540 ± 0.02 ^b^	0.608 ± 0.01 ^a^
Pro	1.175 ± 0.08 ^d^	1.214 ± 0.07 ^cd^	1.259 ± 0.04 ^bcd^	1.280 ± 0.08 ^bcd^	1.303 ± 0.01 ^bc^	1.357 ± 0.05 ^b^	1.531 ± 0.04 ^a^
TAA	10.634 ± 0.30 ^c^	11.129 ± 0.31 ^c^	13.097 ± 0.44 ^b^	13.513 ± 0.38 ^b^	13.709 ± 0.37 ^b^	13.354 ± 0.16 ^b^	14.987 ± 0.34 ^a^
EAA	2.251 ± 0.08 ^e^	2.442 ± 0.12 ^d^	2.928 ± 0.05 ^c^	3.120 ± 0.10 ^b^	3.215 ± 0.14 ^b^	3.153 ± 0.07 ^b^	3.546 ± 0.10 ^a^
NEAA	8.383 ± 0.22 ^c^	8.687 ± 0.19 ^c^	10.169 ± 0.38 ^b^	10.393 ± 0.29 ^b^	10.494 ± 0.24 ^b^	10.201 ± 0.09 ^b^	11.441 ± 0.23 ^a^

Values in the same column with different letters are significantly different (*p* < 0.05).

## Data Availability

The original contributions presented in this study are included in the article. Further inquiries can be directed to the corresponding author.
